# Large common bile duct stone: a case report

**DOI:** 10.1093/jscr/rjaf264

**Published:** 2025-05-02

**Authors:** Sadiq Husain, Yogesh Rawat, Mohd Sadik Akhtar, Sarthak Kulshrestha

**Affiliations:** Department of Surgery, JNMCH, AMU, Medical Road, Zakariya Market, Aligarh 202002, Uttar Pradesh, India; Department of Surgery, JNMCH, AMU, Medical Road, Zakariya Market, Aligarh 202002, Uttar Pradesh, India; Department of Surgery, JNMCH, AMU, Medical Road, Zakariya Market, Aligarh 202002, Uttar Pradesh, India; Department of Surgery, JNMCH, AMU, Medical Road, Zakariya Market, Aligarh 202002, Uttar Pradesh, India

**Keywords:** choledocholithiasis, common bile duct stone, ERCP, biliary obstruction, open CBD exploration, T-tube placement

## Abstract

Choledocholithiasis, the presence of stones within the common bile duct (CBD), poses significant therapeutic challenges, especially when stones exceed 15 mm in diameter. Large CBD stones can lead to complications such as biliary obstruction, cholangitis, and pancreatitis. Endoscopic retrograde cholangiopancreatography (ERCP) is the first-line treatment; however, surgical intervention may be required for refractory cases. We present a case of a 50-year-old female with a giant CBD stone measuring 6 × 4 × 4 cm, managed successfully via open CBD exploration and T-tube placement after failed endoscopic extraction. This case underscores the importance of tailored management strategies for complex biliary stone disease. While ERCP remains the gold standard, open CBD exploration remains a viable option for large, impacted stones.

## Introduction

Choledocholithiasis, defined as the presence of calculi within the common bile duct (CBD), is a common yet potentially serious hepatobiliary condition [1]. Large CBD stones, typically exceeding 15 mm in diameter, present significant therapeutic challenges due to their size and associated complications, including biliary obstruction, cholangitis, and pancreatitis. The etiology is multifactorial, with risk factors including gallstone disease, bile stasis, and infections. Endoscopic retrograde cholangiopancreatography (ERCP) with lithotripsy remains the primary treatment modality, although laparoscopic or open surgical interventions may be necessary in refractory cases.

Here, we report a case of a giant CBD stone measuring 6 × 4 × 4 cm. Notably, some of the largest reported CBD stones in the literature measured 7.5 × 4.0 × 4.0 cm and 3.8 × 2.5 × 1.2 cm [2].

## Case report

A 50-year-old female presented with a 1-year history of right upper quadrant (RUQ) pain, accompanied by recurrent episodes of vomiting and intermittent fever. The patient also reported yellowish discoloration of the eyes 5 months prior. There was no history of hypertension, diabetes mellitus, tuberculosis, or thyroid disorders.

On physical examination, tenderness was noted in the RUQ, with no evidence of organomegaly. Laboratory investigations revealed: hemoglobin: 12 g/dl, white blood cell count: 8000/mm^3^, random blood glucose: 128 mg/dl, serum creatinine: 0.7 mg/dl, liver function tests: alanine aminotransferase (ALT): 281 U/L, aspartate aminotransferse (AST): 170 U/L, alkaline phosphatase (ALP): 771 U/L, total bilirubin: 2.1 mg/dl (direct: 1.6 mg/dl), serum amylase: 45 U/L, serum lipase: 55 U/L.

Radiological imaging revealed a contracted gallbladder with multiple calculi within the lumen, a wall thickness of 3 mm, and choledocholithiasis with a dilated CBD and intrahepatic biliary radicals.

**Figure 1:**
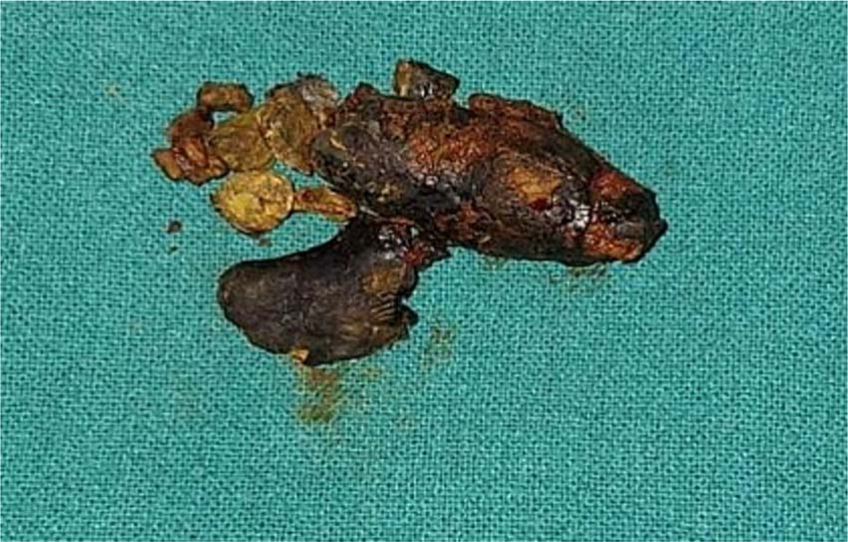
Intraoperative view of the giant common bile duct (CBD) stone measuring 6 × 4 × 4 cm, extending into the cystic duct and common hepatic duct.

The patient was scheduled for an open cholecystectomy with open CBD exploration. Intraoperatively, the gallbladder was found to be contracted and densely adherent to surrounding structures, with a dilated cystic duct and CBD (measuring 4 cm in diameter). Palpation of the CBD revealed a large, hard stone. The gallbladder, which contained multiple small calculi, was removed. A linear incision was then made in the CBD, revealing a giant stone measuring 6 × 4 × 4 cm, which extended into both the cystic duct and the common hepatic duct (Fig. 1). The stone was densely adherent to the posterior wall of the CBD and was carefully extracted. A T-tube was subsequently placed, and the CBD was sutured around it.

**Figure 2:**
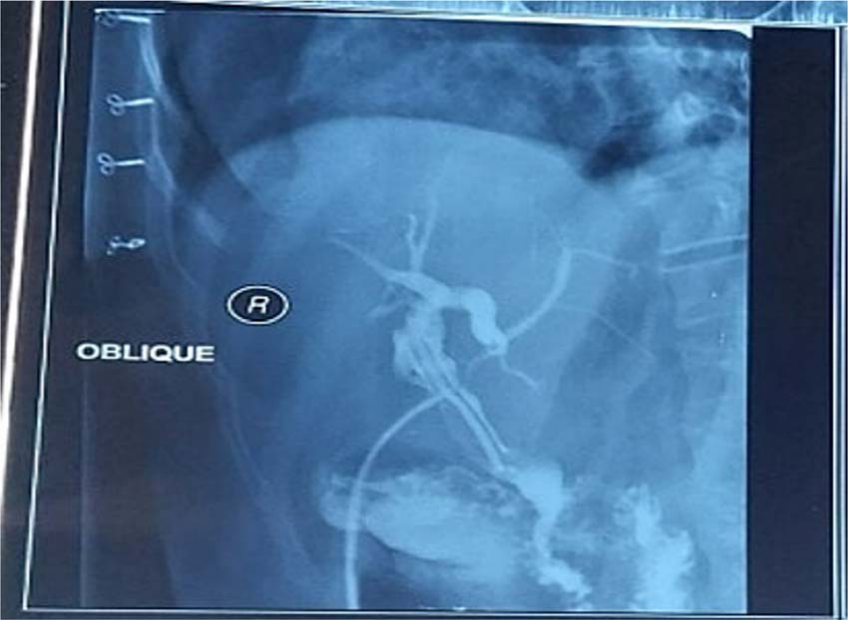
Postoperative T-tube cholangiogram confirming complete stone clearance with no residual filling defects in the CBD.

The postoperative course was uneventful, and the patient was discharged on postoperative Day 5. Three weeks later, a T-tube cholangiogram (Fig. 2) confirmed complete stone clearance, after which the T-tube was removed.

## Discussion

The management of CBD stones has evolved significantly, encompassing endoscopic, surgical, and percutaneous interventions. The choice of treatment depends on stone size, anatomical considerations, patient comorbidities, and institutional expertise.

ERCP is the first-line treatment for CBD stones, particularly in patients without concomitant gallbladder disease requiring surgery. The procedure involves CBD cannulation via the ampulla of Vater, followed by sphincterotomy and stone extraction using balloon catheters or baskets. The success rate of ERCP for stone clearance exceeds 90% in experienced centers, although there is a risk of post-ERCP pancreatitis [3]. For large CBD stones (>15 mm), endoscopic sphincterotomy combined with large balloon dilation enhances stone extraction by widening the biliary orifice [4]. When conventional extraction methods fail, lithotripsy (mechanical, electrohydraulic, or laser) can be employed to fragment large stones, facilitating their removal [5].

Laparoscopic CBD exploration is a viable alternative to ERCP, particularly in patients undergoing cholecystectomy for gallbladder stones. It allows for simultaneous stone clearance and gallbladder removal, reducing the need for postoperative ERCP [6]. However, it requires specialized surgical expertise and equipment. Open CBD exploration remains an option for cases with complex biliary anatomy, impacted stones, or failure of endoscopic approaches. The procedure is often complemented by T-tube placement for postoperative bile drainage, followed by a T-tube cholangiogram 7–10 days later to assess ductal patency and rule out residual stones [6].

Percutaneous transhepatic biliary drainage (PTBD) or percutaneous choledochoscopy can be considered in patients who are poor candidates for ERCP or surgery. PTBD serves as a bridge to definitive therapy, especially in cases complicated by cholangitis [5].

Large CBD stones may mimic choledochal cysts, congenital bile duct anomalies characterized by cystic dilation and presenting with jaundice, pain, and a palpable mass. Magnetic resonance cholangiopancreatography can differentiate the two conditions: choledochal cysts exhibit cystic dilation without intraluminal filling defects, whereas large CBD stones appear as filling defects with proximal ductal dilation. Ultrasonography typically shows an echogenic shadow in CBD stones, whereas choledochal cysts appear as an anechoic dilation [7]. While choledochal cysts require surgical excision, CBD stones are managed via ERCP or surgical extraction [8].

## Conclusion

This case highlights the successful surgical management of a giant CBD stone measuring 6 × 4 × 4 cm, which was refractory to endoscopic extraction. While ERCP remains the gold standard for CBD stone clearance, open CBD exploration with T-tube placement remains a viable option in select cases, particularly when faced with large, impacted stones. Advances in endoscopic, surgical, and percutaneous techniques continue to refine the management strategies for complex biliary stone disease.
